# Salidroside Protects against MPP+-Induced Neuronal Injury through DJ-1-Nrf2 Antioxidant Pathway

**DOI:** 10.1155/2017/5398542

**Published:** 2017-09-28

**Authors:** Leitao Wu, Hang Xu, Liang Cao, Tao Li, Ruru Li, Yang Feng, Jianzong Chen, Jing Ma

**Affiliations:** ^1^Research Center of Traditional Chinese Medicine, Xijing Hospital, Fourth Military Medical University, Xi'an 710032, China; ^2^Department of Pharmacy, Xijing Hospital, Fourth Military Medical University, 169 West Changle Road, Xi'an 710032, China; ^3^Department of Chinese Medicine, Xijing Hospital, Fourth Military Medical University, 169 West Changle Road, Xi'an 710032, China

## Abstract

Parkinson's disease (PD) is the second most common neurodegenerative disorder. We have found that salidroside (Sal) exhibited neuroprotective effects against MPP+ toxicity. However, the molecular mechanism is not fully understood. In this study, we found that Sal significantly prevented MPP+-induced decrease of mRNA and protein expression of Nrf2, GCLc, SOD1, and SOD2 in SH-SY5Y cells. Moreover, silencing of Nrf2 significantly inhibited Sal-induced increase in mRNA and protein expression of GCLc, SOD1, and SOD2. But Nrf2 silence did not significantly impact Sal-exhibited effects on DJ-1 expression. Silencing of Nrf2 significantly suppressed the decrease of apoptosis induced by Sal in MPP+-treated SH-SY5Y cells. Sal significantly prevented MPP+-induced decrease of the mRNA and protein expression of DJ-1 in SH-SY5Y cells. Moreover, silencing of DJ-1 significantly inhibited Sal-induced increase in mRNA and protein expression of Nrf2, GCLc, SOD1, and SOD2 in MPP+-treated SH-SY5Y cells. These results indicated that DJ-1 was an upstream regulator of Nrf2 in the neuroprotective effects of Sal. Furthermore, silencing of DJ-1 significantly suppressed the decrease of apoptosis induced by Sal in MPP+-treated SH-SY5Y cells. In conclusion, Sal prevented MPP+-induced neurotoxicity through upregulation of DJ-1-Nrf2-antioxidant pathway. Our findings provide novel insights into the neuroprotective effects of Sal against PD.

## 1. Introduction

Parkinson's disease (PD) is the second most common neurodegenerative disorder, affecting more than 1% of humans over 60 years of age [1, 2]. PD is attributed to loss of dopaminergic (DA) neurons in the substantia nigra pars compacta (SNpc) and characterized by the presence of ubiquitinated alpha-synuclein- (*α*-syn-) containing cytoplasmic inclusions called Lewy bodies in surviving SNpc neurons [3, 4]. Although the accurate mechanism of PD is not known, oxidative injury and associated apoptotic cell death are believed to play an important role [2, 5, 6]. Large quantity of studies has shown that antioxidant intervention is effective for the treatment of PD and other neurodegenerative diseases [6]. However, clinical therapy of PD is limited by shortage of validated drugs [7, 8].

**Figure 1:**
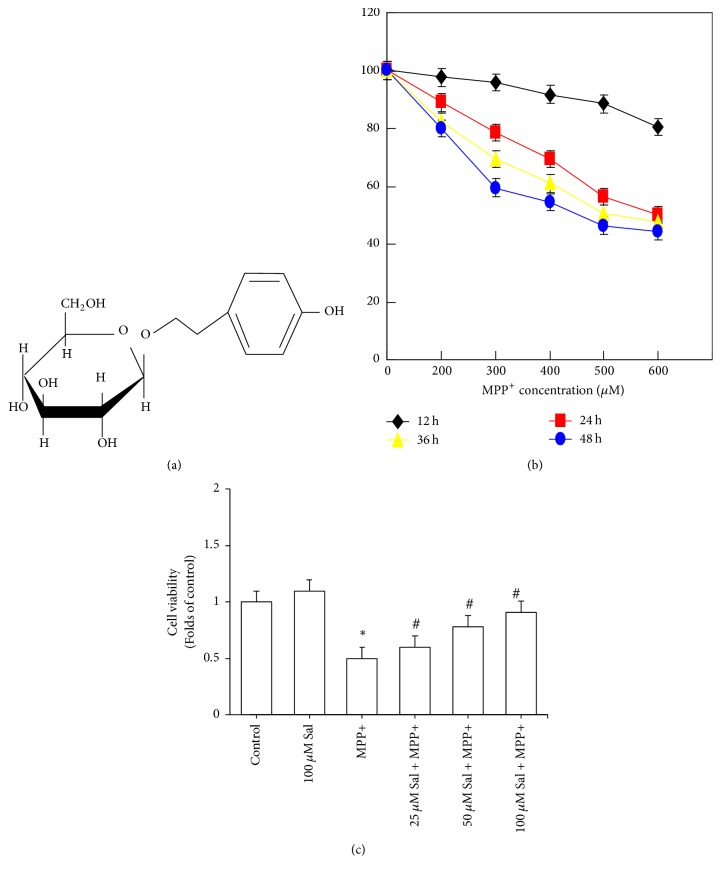
*Effect of Sal on cell viability in MPP+-treated SH-SY5Y cells.* SH-SY5Y cells were pretreated with 25–100 *μ*M Sal for 24 h and then exposed to 500 *μ*M MPP+ for an additional 24 h. At the end, cell viability was determined by MTT assay. ^*∗*^*p* < 0.05, compared with control. ^#^*p* < 0.05, compared with MPP+ treated cells.

Salidroside (p-hydroxyphenethyl-b-D-glucoside; C14H20O7) (Sal) is a major component extracted from* Rhodiola rosea* L., which is a plant popular in traditional medicine in Asian and Eastern European countries (Figure 1(a)). It is shown that Sal possesses a variety of pharmacological activities, including antioxidant, antiaging, antitumor, antihypoxic and neuroprotective effects [9–13]. Our previous studies have shown that Sal protects against apoptosis in SH-SY5Y cells by inhibiting the NO pathway [14]. We also show that Sal protects against PD in mouse model through ROS-NO-related mitochondrion pathway [15]. These results suggest that the antioxidant activity of Sal plays a pivotal role in the neuroprotective effects against PD. However, the mechanism underlying the neuroprotective effects of Sal is still far from being completely understood.

MPTP- (1-methyl-4-phenyl-1,2,5,6- tetrahydropyridine-) induced neuronal injury in neuron cells and neurodegenerative disorder in animals are usually used for the study of the pathogenesis and treatment of PD [16–18]. The neurotoxin MPTP causes neurotoxicity through the active metabolite MPP+ (1-methyl-4-phenylpyridinium). MPP+ could be selectively taken up by dopaminergic neurons via the dopamine transporter of the plasma membrane [19]. MPP+ results in neuronal loss in substantia nigra and striatal dopamine (DA) depletion, leading to PD-like behavioral impairments [20, 21]. Moreover, oxidative stress is involved in MPP+-associated PD-like neuronal injury [22, 23].

DJ-1 is a novel antioxidant protein and abnormal regulation of DJ-1 is closely related to the development of PD [24–27]. In the present study, we aimed to investigate whether the neuroprotective activities of Sal against PD involve the regulation of DJ-1, using SH-SY5Y cells, a clonal human neuroblastoma cell line. We found that Sal protects against MPP+-induced apoptotic cell death in SH-SY5Y cells through regulation of DJ-1-nuclear factor- (erythroid-derived 2-) like 2 (Nrf2) pathway.

## 2. Materials and Methods

### 2.1. Chemicals and Materials

*β*-actin and Nrf2 antibodies were purchased from Santa Cruz Biotechnology Inc. (Santa Cruz). DJ-1, GCLc, SOD1, and SOD2 antibodies were obtained from Cell Signaling Technology (CST, USA). Hoechst 33258 and MPP^+^ were obtained from Sigma (St. Louis, MO). Sal was obtained from the National Institute for the Control of Pharmaceutical and Biological Products, Xi'an, China.

### 2.2. Cell Culture and Transfection

SH-SY5Y cells were purchased from American Type Culture Collection (ATCC, USA) and cultured in Dulbecco's modified Eagle medium (Gibco, Gaithersburg, MD, USA) supplemented with 10% heat-inactivated horse serum (Gibco), 5% heat-inactivated fetal calf serum (Gibco), 100 IU/ml penicillin, and 100 mg/ml streptomycin in a humidified incubator containing 5% CO_2_ at 37°C. SH-SY5Y cells were treated with 50 ng/ml 2.5S nerve growth factor (Promega, Madison, WI, USA) for 9 days to induce differentiation. Cells were plated in 6-well plates and passaged at 60–70% confluence. In the experiments, cells were pretreated with 25–100 *μ*M Sal for 24 h and then exposed to 500 *μ*M MPP+ for an additional 24 h. In some experiments, cells were transfected with siNrf2 or siDJ-1 (Santa Cruz Biotechnology) using lipofectamine 2000 transfection reagent 2–4 days before the treatment of Sal and MPP+.

### 2.3. Cell Viability

SH-SY5Y cells were seeded in 96-well plates at 1 × 10^4^ cells per well. After the treatment with Sal and MPP+, cell viability was measured by the 3-(4,5-dimethylthiazol-2-yl)-2,5-diphenyltetrazolium bromide (MTT; Sigma Aldrich) assay. Briefly, cells were incubated with 500 *μ*g/ml MTT at 37°C for 4 h. After that, the medium was removed and 150 *μ*l dimethyl sulfoxide (DMSO) was added and shaking was conducted for 10 min. Absorbance was measured at 570 nm in a microplate reader (BioRad, USA) and the results were expressed as folds of control.

### 2.4. Determination of Cell Death

Cell death was evaluated using an FITC-labeled Annexin V and propidium iodide (PI) assay kit (Sigma). In brief, after the treatment, cells were harvested and centrifuged and then resuspended in binding buffer at a concentration of 1 × 10^6^ cells/ml. Then, the mixture was added with 5 *μ*l of 20 *μ*g/ml Annexin V and 50 *μ*g/ml PI and incubated in a humidified incubator for 15 min. After the binding, cell death was analyzed by a flow cytometer (BD Biosciences, CA, USA).

### 2.5. Evaluation of Cell Morphology

SH-SY5Y cells were plated on coverslips precoated with poly-L-lysine in 24-well plates and treated by Sal and MPP+ as mentioned above. After the treatment, cells were fixed with 4% paraformaldehyde for 30 min, washed with PBS, and then incubated with Hoechst 33258 (3 *μ*g/ml) for 30 min at room temperature in the dark. After rinsing with PBS, fluorescence was assessed using a fluorescence microscope (Olympus, Japan).

### 2.6. Reverse Transcriptase-Polymerase Chain Reaction Analysis (RT-PCR)

mRNA was isolated from the cells using trizol-reagent (Invitrogen, USA) and the quality of isolated RNA was checked by 1.2% formaldehyde. The RT-PCR reaction used a template cDNA followed by PCR amplification with Taq DNA polymerase in the same tube. PCR products were analyzed by 1.5% agarose gel electrophoresis, stained with ethidium bromide, and photographed under ultraviolet light.

### 2.7. Western Blot Analysis

Cells were lysed in lysis buffer [50 mM Tris-Cl (pH 8.0), 150 mM NaCl, 0.5 mM MgCl_2_, 10% glycerol, 1% Triton X-100, and 0.1% SDS] with protease inhibitor cocktail (Roche Diagnostics, Switzerland) on ice for 20 min. The lysates were centrifuged for 20 min at 20,000*g*. Protein concentration was determined using BCA method (Thermo Fisher Scientific, USA) and lysates were mixed with loading buffer. The protein mixtures were separated by SDS-PAGE and transferred to PVDF membranes. The membranes were incubated with primary antibodies and then incubated with HRP-conjugated secondary antibody (Thermo Fisher Scientific, USA). Bands were visualized by chemiluminescence reaction using an ECL detection system (Thermo Fisher Scientific, USA), followed by capture using BioRad Imaging Systems (BIORAD, USA).

### 2.8. Evaluation of Oxidative Stress

For the determination of ROS, cells were incubated with 1 *μ*M 5-(and-6)-chloromethyl-2′,7′-dichlorodihydrofluorescein diacetate and acetyl ester (Thermo Fisher) prepared in Hank's Balanced Salt Solution for 20 min at 37°C and then washed for three times and scanned using a microplate fluorometer (Tecan) with Ex at 510 nm/Em at 580 nm and Ex at 495 nm/Em at 520 nm, respectively. Glutathione was measured using a luminescence-based system (GSH Assay, Promega, Madison, WI, USA). Cells were washed with Hank's Balanced Salt Solution and lysed with lysis regents. The GSH level was expressed as in relative light units (RLU)/mg protein.

### 2.9. Statistical Analysis

All assays were carried out in three independent experiments and the results were expressed as the mean ± SEM and analyzed by GraphPad Prism software. The statistical significance of differences among more than two groups was analyzed by one-way analysis of variance (ANOVA) followed by Dunnett's *t*-test for multiple comparisons. The data were deemed to be statistically significant when the* p* value was less than 0.05.

## 3. Results

### 3.1. Sal Protects against MPP+-Induced Cytotoxicity in SH-SY5Y Cells

**Figure 2:**
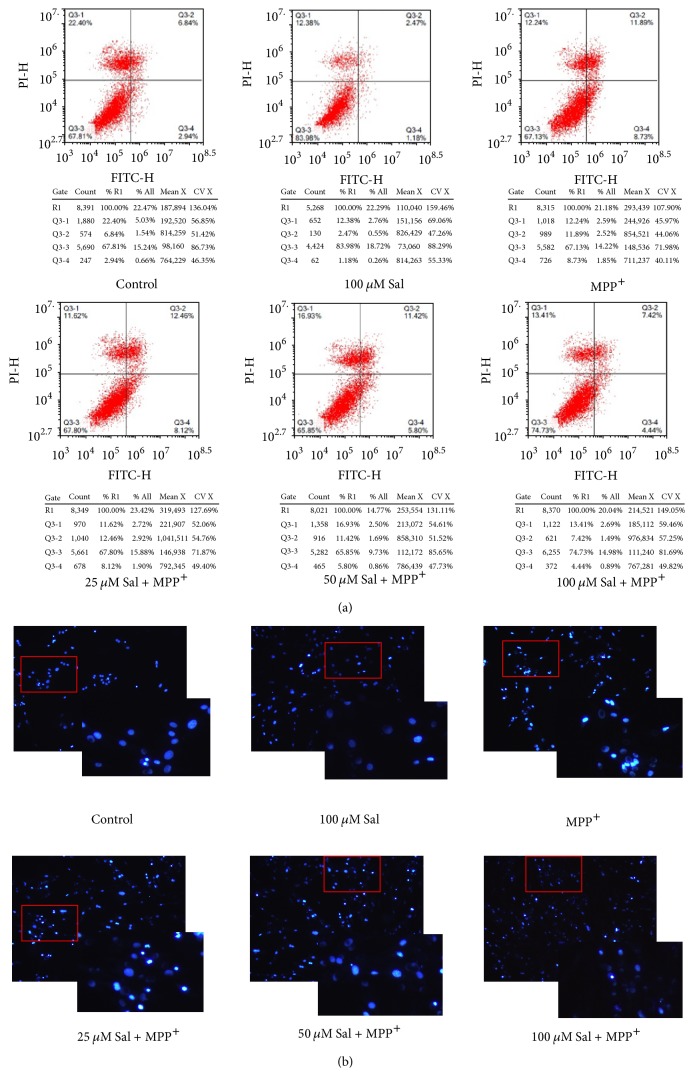
*Effect of Sal on cell death in MPP+-treated SH-SY5Y cells.* SH-SY5Y cells were pretreated with 25–100 *μ*M Sal for 24 h and then exposed to 500 *μ*M MPP+ for an additional 24 h. At the end, cell death was determined using Annexin V/PI assay kit and analyzed by flow cytometry (a). Cells were also stained with Hoechst to observe the morphological changes of nuclei (b).

SH-SY5Y cells are usually used as a neuron model for studies of MPP+ neurotoxicity and PD [28]. In the present study, we evaluated the neuroprotective effect of Sal using SH-SY5Y cells. Cells were exposed to 0–600 *μ*M MPP+ for 12–48 h and the results showed that MPP+ resulted in a significant decrease of cell viability in a concentration and time-dependent manner (Figure 1(b)). Cells were pretreated with 25–100 *μ*M Sal for 24 h and then exposed to 500 *μ*M MPP+ for an additional 24 h. We showed that Sal concentration-dependently prevented MPP+-induced decrease of cell viability (Figure 1(c)). Annexin V/PI staining is a common method for the detection of apoptotic cell. We found that Sal significantly decreased the number of Annexin V/PI-stained cells treated by MPP+ which was in a concentration-dependent manner (Figure 2(a)). Apoptotic cell could also be morphologically evaluated by Hoechst staining. In Hoechst staining, apoptotic cells are characterized by reduced nuclear size, chromatin condensation, intense fluorescence, and nuclear fragmentation. We showed that Sal notably inhibited MPP+-induced increase of chromatin condensation, intense fluorescence, and nuclear fragmentation in SH-SY5Y cells (Figure 2(b)). These results indicated that Sal protected against MPP+-induced cytotoxicity in SH-SY5Y cells.

### 3.2. Sal Protects against MPP+-Induced Changes of Antioxidant Protein Expression in SH-SY5Y Cells

**Figure 3:**
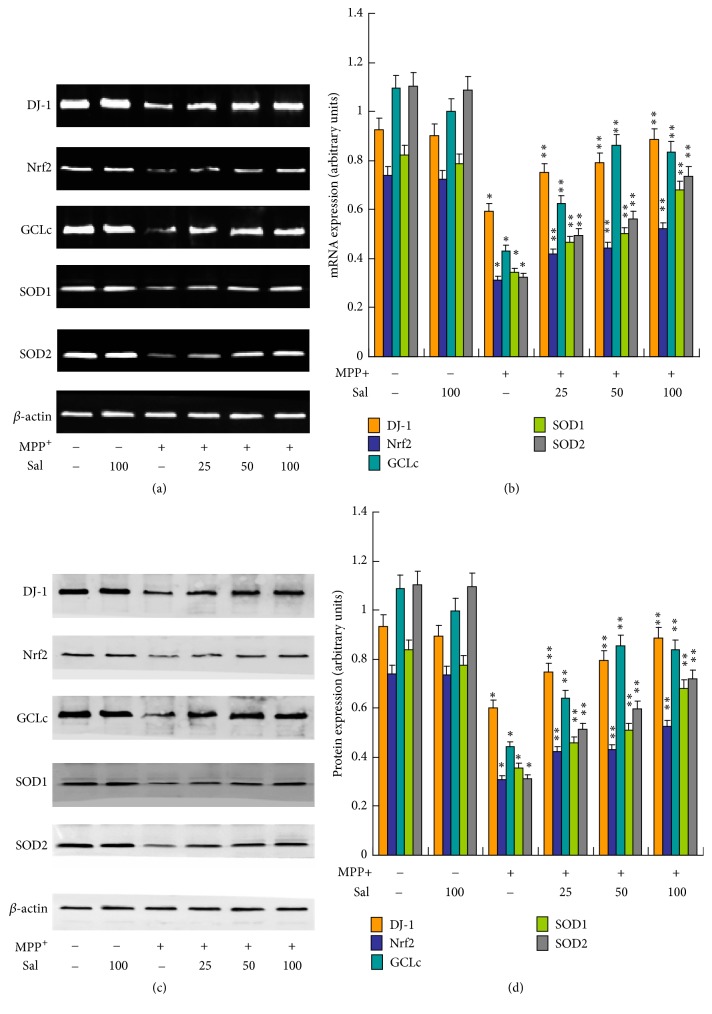
*Effect of Sal on key antioxidant regulator expression in MPP+-treated SH-SY5Y cells.* SH-SY5Y cells were pretreated with 25–100 *μ*M Sal for 24 h and then exposed to 500 *μ*M MPP+ for an additional 24 h. ((a) and (b)) mRNA expression of DJ-1, Nrf2, GCLc, SOD1, and SOD2 was determined and statistical analysis was showed. ((c) and (d)) Protein expression of DJ-1, Nrf2, GCLc, SOD1, and SOD2 was determined and statistical analysis was showed. ^*∗*^*p* < 0.05, compared with control. ^*∗∗*^*p* < 0.05, compared with MPP^+^.

To further examine the mechanism of Sal-exhibited neuroprotective effects, we examined the effect of Sal on the expression of key antioxidant proteins, including DJ-1, Nrf2, glutamate-cysteine ligase catalytic subunit (GCLc), superoxide dismutase 1 (SOD1), and SOD2. The results showed that MPP+ induced a significant decrease of the mRNA (Figures 3(a) and 3(b)) and protein (Figures 3(c) and 3(d)) expression of DJ-1, Nrf2, GCLc, SOD1, and SOD2. The treatment of Sal significantly prevented MPP+-induced decrease of the mRNA (Figures 3(a) and 3(b)) and protein (Figures 3(c) and 3(d)) expression of DJ-1, Nrf2, GCLc, SOD1, and SOD2 in SH-SY5Y cells. The results indicated that Sal exhibited protective effects against MPP+-induced imbalance of redox state through regulation of key antioxidant proteins.

### 3.3. Upregulation of Nrf2 Is Involved in the Neuroprotective Effects of Sal against MPP+ Cytotoxicity

**Figure 4:**
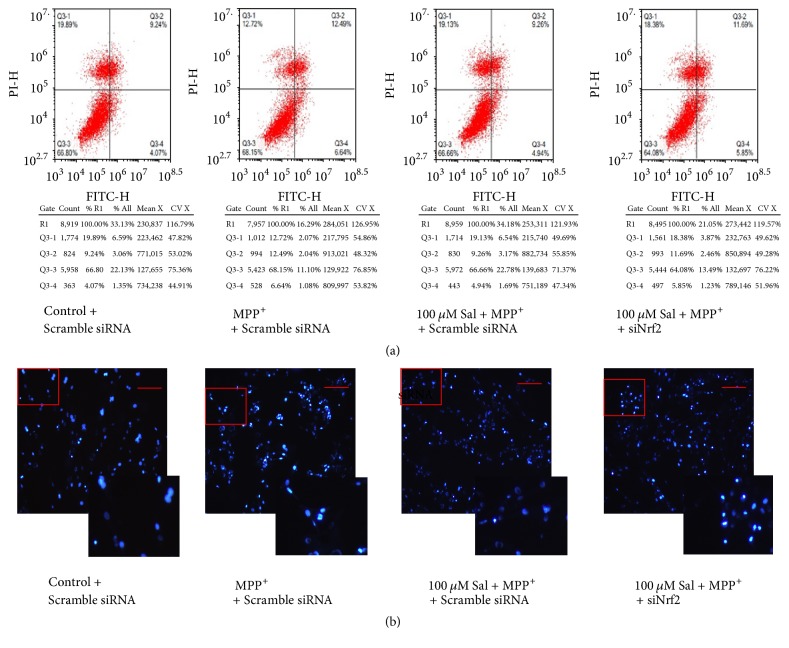
*Role of Nrf2 in Sal-induced effects on cell death in MPP+-treated SH-SY5Y cells.* SH-SY5Y cells were transfected with siNrf2, pretreated with 25–100 *μ*M Sal for 24 h, and then exposed to 500 *μ*M MPP+ for an additional 24 h. At the end, cell death was determined using Annexin V/PI assay kit and analyzed by flow cytometry (a). Cells were also stained with Hoechst to observe the morphological changes of nuclei (b).

**Figure 5:**
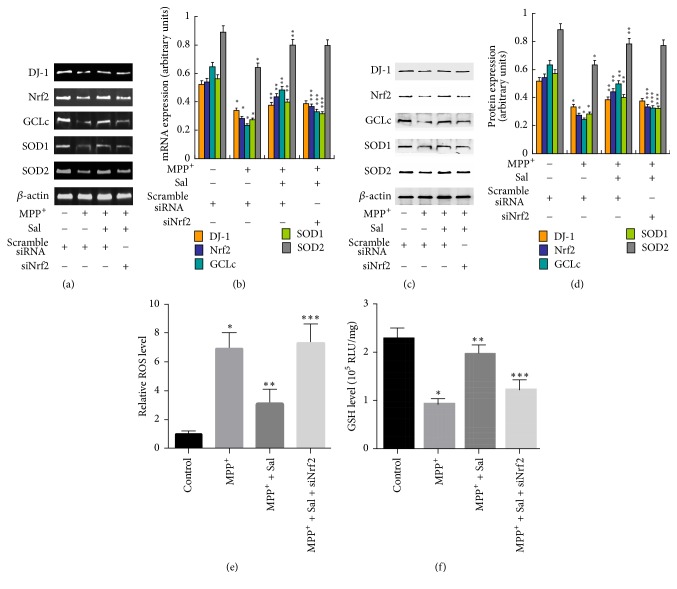
*Role of Nrf2 in Sal-induced effects key antioxidant regulator expression in MPP+-treated SH-SY5Y cells.* SH-SY5Y cells were transfected with siNrf2, pretreated with 25–100 *μ*M Sal for 24 h, and then exposed to 500 *μ*M MPP+ for an additional 24 h. ((a) and (b)) mRNA expression of DJ-1, Nrf2, GCLc, SOD1, and SOD2 was determined and statistical analysis was showed. ((c) and (d)) Protein expression of DJ-1, Nrf2, GCLc, SOD1, and SOD2 was determined and statistical analysis was showed. (e) ROS level was determined using DCFH-DA staining. (f) GSH level was determined by an assay kit. ^*∗*^*p* < 0.05, compared with control. ^*∗∗*^*p* < 0.05, compared with MPP^+^. ^*∗∗∗*^*p* < 0.05, compared with Sal.

To examine whether regulation of Nrf2 was involved in the neuroprotective effects of Sal against MPP+ cytotoxicity, SH-SY5Y cells were transfected with siNrf2. We showed that silencing of Nrf2 significantly suppressed the decrease of Annexin V/PI-stained cells induced by Sal in MPP+-treated SH-SY5Y cells (Figure 4(a)). Sal-induced decrease of chromatin condensation, intense fluorescence, and nuclear fragmentation in MPP+-treated cells were inhibited by siNrf2 (Figure 4(b)). Moreover, silencing of Nrf2 significantly inhibited Sal-induced increase in mRNA (Figures 5(a) and 5(b)) and protein (Figures 5(c) and 5(d)) expression of Nrf2, GCLc, SOD1, and SOD2 in MPP+-treated SH-SY5Y cells. However, Nrf2 silence did not significantly affect Sal-exhibited effects on DJ-1 expression (Figures 5(a), 5(b), 5(c), and 5(d)). Moreover, Sal inhibited MPP+-induced increase of reactive oxygen species (ROS) (Figure 5(e)) and decrease of glutathione (GSH) level (Figure 5(f)). Silencing of Nrf2 significantly inhibited Sal-induced decrease ROS level and increase of GSH level in MPP+-treated cells (Figures 5(e) and 5(f)). The results indicated that Nrf2-regulated antioxidant enzymes were involved in the neuroprotective effects of Sal against MPP+ toxicity.

### 3.4. Upregulation of DJ-1 Is Involved in the Neuroprotective Effects of Sal against MPP+ Cytotoxicity

**Figure 6:**
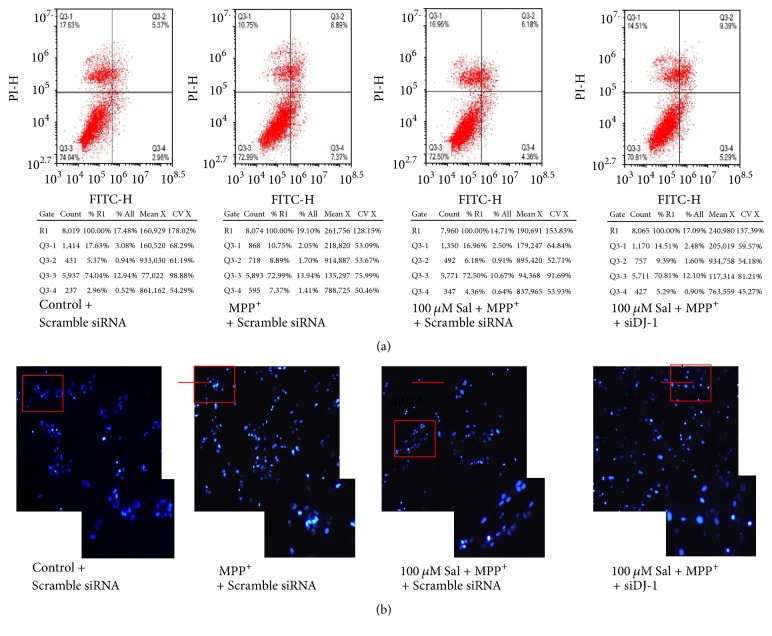
*Role of Nrf2 in Sal-induced effects on cell death in MPP+-treated SH-SY5Y cells.* SH-SY5Y cells were transfected with siDJ-1, pretreated with 25–100 *μ*M Sal for 24 h, and then exposed to 500 *μ*M MPP+ for an additional 24 h. At the end, cell death was determined using Annexin V/PI assay kit and analyzed by flow cytometry (a). Cells were also stained with Hoechst to observe the morphological changes of nuclei (b).

**Figure 7:**
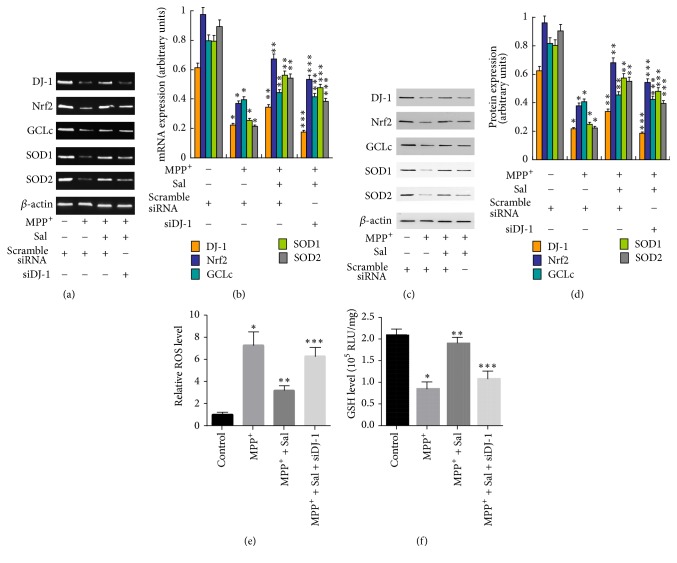
*Role of Nrf2 in Sal-induced effects on key antioxidant regulator expression in MPP+-treated SH-SY5Y cells.* H-SY5Y cells were transfected with siDJ-1, pretreated with 25–100 *μ*M Sal for 24 h, and then exposed to 500 *μ*M MPP+ for an additional 24 h. ((a) and (b)) mRNA expression of DJ-1, Nrf2, GCLc, SOD1, and SOD2 was determined and statistical analysis was showed. ((c) and (d)) Protein expression of DJ-1, Nrf2, GCLc, SOD1, and SOD2 was determined and statistical analysis was showed. (e) ROS level was determined using DCFH-DA staining. (f) GSH level was determined by an assay kit. ^*∗*^*p* < 0.05, compared with control. ^*∗∗*^*p* < 0.05, compared with MPP^+^. ^*∗∗∗*^*p* < 0.05, compared with Sal.

SH-SY5Y cells were further transfected with siDJ-1 to examine whether regulation of DJ-1 was involved in the neuroprotective effects of Sal against MPP+ cytotoxicity. The results showed that silencing of DJ-1 significantly suppressed the decrease of Annexin V/PI-stained cells induced by Sal in MPP+-treated SH-SY5Y cells (Figure 6(a)). Moreover, Sal-induced decrease of chromatin condensation, intense fluorescence, and nuclear fragmentation in MPP+-treated cells were inhibited by siDJ-1 (Figure 6(b)). Furthermore, silencing of DJ-1 significantly inhibited Sal-induced increase in mRNA (Figures 7(a) and 7(b)) and protein (Figures 7(c) and 7(d)) expression of DJ-1, Nrf2, GCLc, SOD1, and SOD2 in MPP+-treated SH-SY5Y cells. Moreover, silencing of DJ-1 significantly inhibited Sal-induced decrease of ROS level and increase of GSH level in MPP+-treated cells (Figures 7(e) and 7(f)). The results indicated that DJ-1 was an upstream regulator of Nrf2-antioxidant enzymes, which were involved in the neuroprotective effects of Sal against MPP+ toxicity.

## 4. Discussion

Our previous studies have found that Sal exhibited neuroprotective effects against MPP+ toxicity in vitro and in vivo [14, 15, 29]. However, the molecular mechanism underlying this beneficial effect is far from being completely understood. We explored the antioxidant mechanism of Sal-possessed neuroprotective effects in SH-SY5Y cells.

Increase of ROS generation and oxidative stress is considered to be a common pathway in the development of PD and its associated diseases [30–32]. It is well known that Nrf2 is central regulator of redox status in various biological processes. Nrf2 can bind with antioxidant response element, controlling the transcription of a battery antioxidant enzymes, including GCL and SOD [33]. SOD functions to catalyze the transition of O^2-^ to oxygen and H_2_O_2_. SOD1 (cytoplasmic copper and zinc SOD, Cu/ZnSOD) and SOD2 (mitochondrial manganese SOD, MnSOD) are the main types of SOD in organisms. GCLc is the catalytic subunit of GCL, which is the rate-limiting enzyme responsible for catalyzing de novo synthesis of glutathione (GSH) from the precursor amino acids cysteine, glutamate, and glycine [34]. GSH is the most important hydrophilic antioxidant and plays a critical role in various cellular processes through direct detoxification or acting as a cofactor. Abnormal expression of Nrf2, GCLc, SOD1, and SOD2 has been reported to be associated with PD and other neurodegenerative diseases [35–40]. In the present study, we tested the possible role of Nrf2 and related antioxidant enzymes in the protective effects of Sal. We found that Sal significantly prevented MPP+-induced decrease of the mRNA and protein expression of Nrf2, GCLc, SOD1, and SOD2 in SH-SY5Y cells. Moreover, silencing of Nrf2 significantly inhibited Sal-induced increase in mRNA and protein expression of GCLc, SOD1, and SOD2 in MPP+-treated SH-SY5Y cells. But Nrf2 silence did not significantly affect Sal-exhibited impacts on DJ-1 expression, indicating that DJ-1 was not a downstream regulator of Nrf2 signaling. Silencing of Nrf2 significantly suppressed the decrease of apoptosis induced by Sal in MPP+-treated SH-SY5Y cells. The results demonstrated that Nrf2-regulated antioxidant enzymes were involved in the neuroprotective effects of Sal.

DJ-1 is a novel antioxidant regulator that could protect cells from oxidative stress and induce Nrf2 expression [41]. Recent evidence has also suggested that abnormal function of DJ-1 plays a role in the pathogenesis of PD [42–44]. In this study, we also examined the role of DJ-1 in neuroprotective effects of Sal. We found that Sal significantly prevented MPP+-induced decrease of the mRNA and protein expression of DJ-1 in SH-SY5Y cells. Moreover, silencing of DJ-1 significantly inhibited Sal-induced increase in mRNA and protein expression of Nrf2, GCLc, SOD1, and SOD2 in MPP+-treated SH-SY5Y cells. These results indicated that DJ-1 was an upstream regulator of Nrf2 in neuroprotective effects of Sal. Furthermore, silencing of DJ-1 significantly suppressed the decrease of apoptosis induced by Sal in MPP+-treated SH-SY5Y cells. The results demonstrated that DJ-1-regulated Nrf2 antioxidant pathway was involved in the neuroprotective effects of Sal.

In conclusion, we found that Sal prevented MPP+-induced neurotoxicity through upregulation of DJ-1-Nrf2-antioxidant pathway. Further studies are needed to investigate whether DJ-1 exhibits a transcriptional regulation of Nrf2 and those antioxidant enzymes or affects Nrf2-antioxidant enzymes through indirect regulation. Overall, our findings provide novel insights into the neuroprotective effects of Sal against PD and associated neurodegenerative diseases.
